# An Integrated Approach for Efficient and Accurate Medicinal Cuscutae Semen Identification

**DOI:** 10.3390/plants9111410

**Published:** 2020-10-22

**Authors:** Inkyu Park, Sungyu Yang, Goya Choi, Byeong Cheol Moon, Jun-Ho Song

**Affiliations:** Herbal Medicine Resources Research Center, Korea Institute of Oriental Medicine, Naju 58245, Korea; pik6885@kiom.re.kr (I.P.); sgyang81@kiom.re.kr (S.Y.); serparas@kiom.re.kr (G.C.)

**Keywords:** barcode primer, *Cuscuta chinensis*, Cuscutae Semen, endosperm, light microscope, morphological identification key, scanning electron microscope, seed morphology, *rbcL* gene

## Abstract

To guarantee the safety and efficacy of herbal medicines, accurate identification and quality evaluation are crucial. The ripe dried seeds of *Cuscuta australis* R.Br. and *C. chinensis* Lam. are known as Cuscutae Semen (CS) and are widely consumed in Northeast Asia; however, the seeds of other species can be misidentified as CS owing to morphological similarities, leading to misuse. In this report, we propose a multilateral strategy combining microscopic techniques with statistical analysis and DNA barcoding using a genus-specific primer to facilitate the identification and authentication of CS. Morphology-based identification using microscopy revealed that the useful diagnostic characteristics included general shape, embryo exudation, hairiness, and testa ornamentation, which were used to develop an effective identification key. In addition, we conducted DNA barcoding-based identification to ensure accurate authentication. A novel DNA barcode primer was produced from the chloroplast *rbcL* gene by comparative analysis using *Cuscuta* chloroplast genome sequences, which allowed four *Cuscuta* species and adulterants to be discriminated completely. Therefore, this investigation overcame the limitations of universal DNA barcodes for *Cuscuta* species with high variability. We believe that this integrated approach will enable CS to be differentiated from other species, thereby improving its quality control and product safety in medicinal markets.

## 1. Introduction

Medicinal plants and plant-derived medicines are commonly used worldwide in traditional Chinese medicine and are recognized as natural alternatives or supplements to synthetic chemicals from a modern pharmacological perspective [1]; however, there have been repeated reports of contamination of herbal products due to inaccurate identification and poor quality control, i.e., substitution, adulteration, and/or admixture of other species, at national [2,3,4], continental [5,6], or global markets [7,8]. As the quality of herbal medicines directly affects treatment efficacy and drug safety, ensuring the quality, safety, and effectiveness of these medicinal materials has become a serious issue [9,10,11,12]. Thus, pharmacovigilance is increasingly important for developing reliable information on the safety of herbal medicines [13,14].

According to the Korean Herbal Pharmacopoeia, the dried ripe seeds of *Cuscuta chinensis* Lam. (Convolvulaceae) are a herbal medicine known as Cuscutae Semen (CS), or “To-Sa-Ja” in Korean [15,16]. However, the Pharmacopoeia of the Democratic People’s Republic of Korea lists seeds from both *C. australis* R.Br. and *C. japonica* Choisy as sources of the same herbal medicine [15,17], while the Pharmacopoeia of the People’s Republic of China and the Taiwan Herbal Pharmacopoeia list seeds from *C. chinensis* and *C. australis* as authentic CS, known as “Tu-Si-Zi” in Chinese [15,18,19]. The differences between these definitions have resulted in the circulation of adulterants or counterfeits in herbal markets [20]; therefore, it is important to obtain information regarding species related to CS.

Modern pharmacological studies have revealed that CS can act in various parts of the body, including the reproductive [21,22] and immune [23,24,25] systems, and exerts biological functions such as anti-oxidant [26,27,28] and anti-cancer [29,30] effects. In addition, some reports have suggested that CS can prevent and treat liver [28,31,32,33,34] and neurodegenerative-related [35] diseases.

Unfortunately, seeds with similar morphological traits from southern regions of Korea are misused as CS. For example, seeds from *Perilla frutescens* (L.) Britton var. *frutescens* and *P. frutescens* var. *crispa* (Thunb.) H.Deane (family Lamiaceae, Labiatae) are often found as adulterants in CS due to their morphological similarities [20,36,37]. Moreover, similar congeneric species such as *C. australis*, *C. japonica*, and *C. pentagona* are also found throughout Korea [38,39].

*Cuscuta* (dodder) species are parasitic vines with reduced or almost absent vegetation and very small floral organs. Due to their peculiar morphology and parasitism, the species have received interest from various fields, including agriculture [40], conservation [41], taxonomy (including morphology) [42,43,44,45], and genomics [46,47,48]. However, no studies have yet investigated the accurate identification and authentication of CS as medicinal seeds among morphologically similar species.

Seeds are commonly used as traditional herbal medicines; however, commercial markets often sell mixed seeds from different species, including adulterants, within a single package, while some authentic medicinal seeds can be difficult to distinguish [49,50]. Therefore, multilateral approaches including microscopic analysis have been developed to authenticate and distinguish genuine species from congeneric species and adulterants such as Lepidii Seu Descurainiae Semen [51] and Pharbitidis Semen [52]. Microscopic authentication of medicinal materials has proven to be a highly reliable and accurate identification tool [8,51,52,53,54]. DNA barcoding has also been recognized as a powerful tool for identifying herbal medicines [55,56] because it is convenient, efficient, and accurate. Therefore, the technique has a wide range of potential applications for identifying adulterants in commercial herbal products [56] and for inputting barcoding sequences into the medicinal database [55,57].

In this study, we aimed to elucidate detailed morphological and micromorphological characteristics of CS and adulterants using microscopic analysis, thereby developing a convenient and effective identification key. In addition, we designed a specific primer to distinguish between *C. chinensis* and congeneric species using DNA barcodes. Together, these data will facilitate the quality control of the valuable medicinal seed, CS.

## 2. Results

### 2.1. Morphological Characteristics

**Figure 1:**
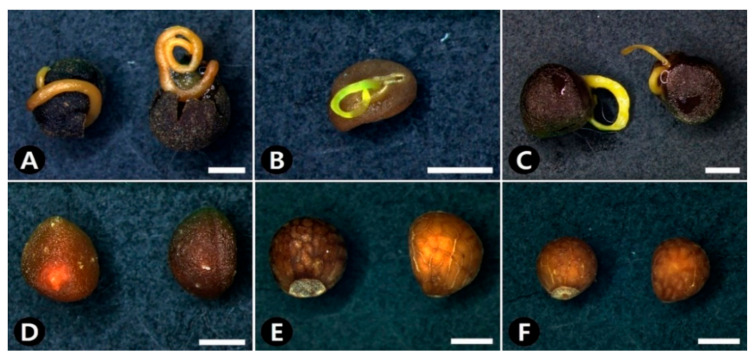
Stereomicrographs showing seed morphology and embryo exudation. (**A**) *Cuscuta australis*. (**B**) *C. chinensis*. (**C**) *C. japonica*. (**D**) *C. pentagona*. (**E**) *Perilla frutescens* var. *frutescens*. (**F**) *P. frutescens* var. *crispa*. Scale bars = 1 mm.

All seeds in the genus *Cuscuta,* including CS, were ovoid in shape and triangular in cross-sectional shape (Figure 1A–D, Table 1); however, the seeds of the two adulterants had a globose to sub-globose shape and a circular to broadly triangular cross-sectional shape (Figure 1E,F, Table 1).

The seeds of the studied species varied from 1.17–3.26 mm in length and 0.98–2.78 mm in width, with *C. australis* having the smallest seeds (average length × width: 1.48 × 1.12 mm) and *C. japonica* having the largest (average length × width: 2.71 × 2.16 mm; Table 1). The seeds were dull brown (200A-D), gray-brown (199A-B, N199A-B, N199A-C, N199C-D), and black (203) in color. In particular, *C. australis*, *C. chinensis*, and *C. japonica* had darker seeds, whereas *C. pentagona*, *P. frutescens* var. *frutescens*, and *P. frutescens* var. *crispa* had lighter seeds (Figure 1, Table 1). In addition, the embryos of *C. australis*, *C. chinensis*, and *C. japonica* exuded from their seeds when placed into boiling water for 10 min (Figure 1A–C, Table 1); however, no changes were observed for *C. pentagona*, *P. frutescens* var. *frutescens*, or *P. frutescens* var. *crispa* seeds following the same treatment (Figure 1D–F, Table 1).

### 2.2. Statistical Analysis of Morphological Characteristics

**Figure 2:**
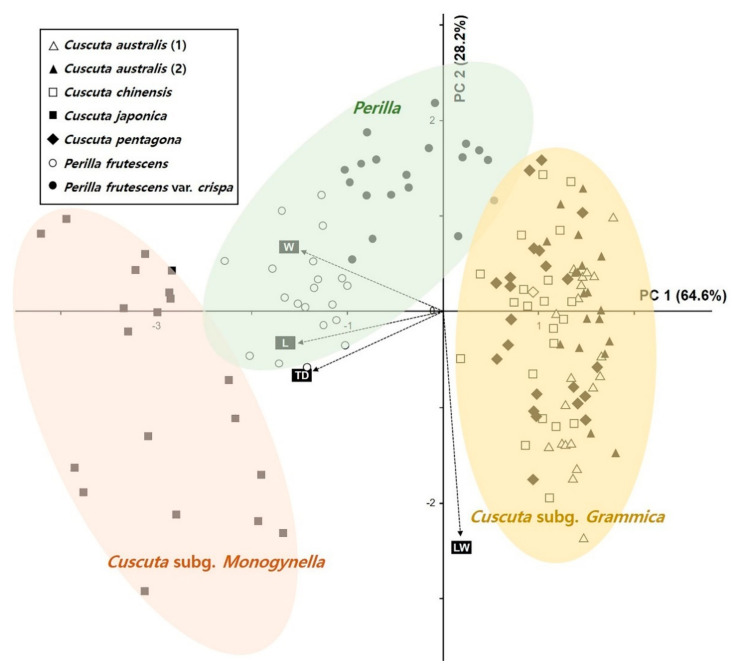
Principal component analysis (PCA) with four quantitative variables for the studied taxa. L, length; LW, L/W; TD, testa diameter; W, width.

Next, we explored the relationships between the quantitative data for each species of seed using principal component analysis (PCA; Figure 2). The first two principal components (PC1 and PC2) explained 92.80% of the total variance, whereas PC1 explained 64.60% of the variance in seed length (L) and width (W) and testa cell (TD) size and PC2 accounted for 28.20% of the variance in the seed size ratio (L/W). The PCA biplot split the operational taxonomic units (OTUs) into three main groups (Figure 2). The OTUs for *C. australis* including commercial CS, *C. chinensis*, and *C. pentagona*, which belong to the subgenus *Grammica*, were grouped on the positive side of the PC1 axis, whereas those for *C. japonica*, which belongs to the subgenus *Monogynella*, were grouped on the negative side of the PC1 axis (Figure 2). The adulterant OTUs (*P. frutescens* vars. *frutescens* and *crispa*) were on the central to positive side of the PC2 axis (Figure 2).

### 2.3. Micromorphological Characteristics

**Figure 3:**
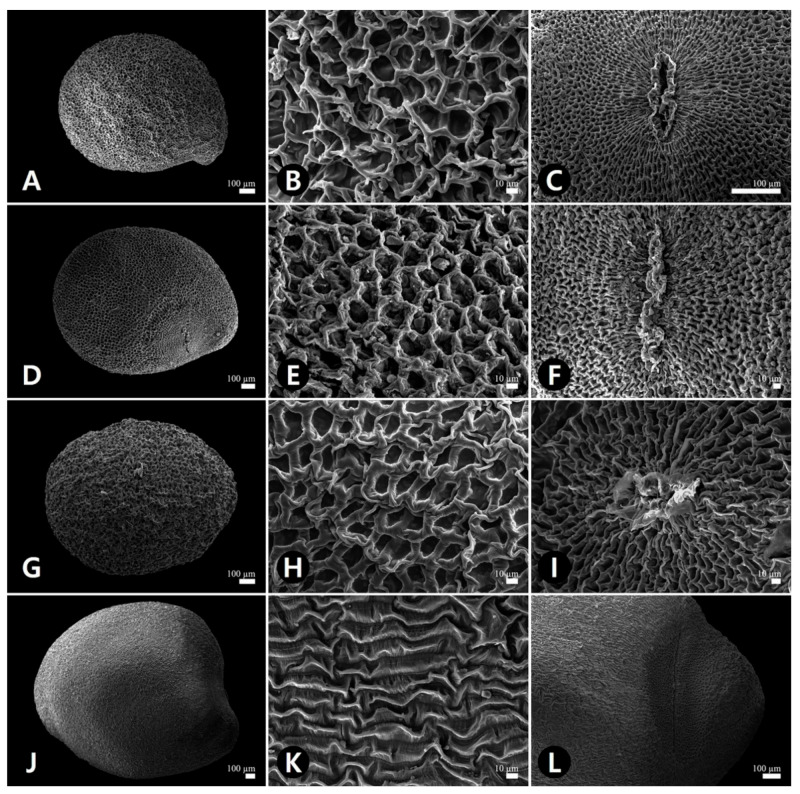
Scanning electron micrographs showing the seed (**A**,**D**,**G**,**J**), detailed testa ornamentation (**B**,**E**,**H**,**K**), and the hilum (**C**,**F**,**I**,**L**). (**A**–**C**) *Cuscuta australis* (1). (**D**–**F**) *C. australis* (2). (**G**–**I**) *C. chinensis*. (**J**–**L**) *C. japonica*.

**Figure 4:**
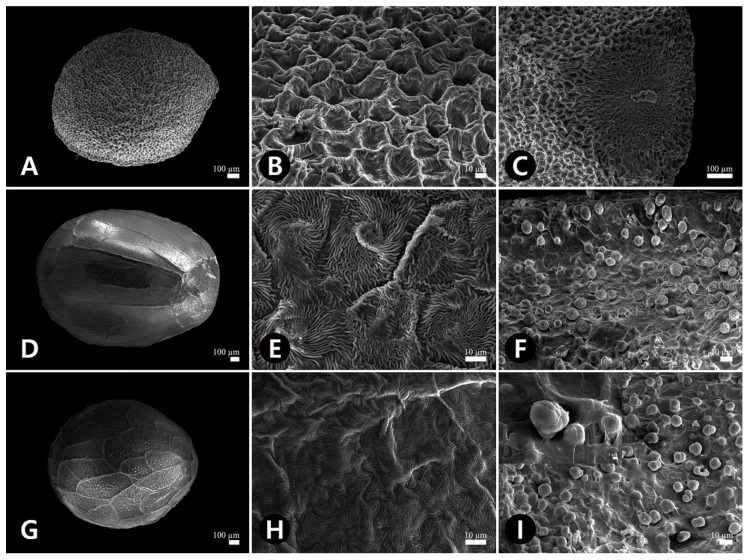
Scanning electron micrographs showing the seed (**A**,**D**,**G**), detailed testa ornamentation (**B**,**E**,**H**), and the hilum (**C**,**F**,**I**). (**A**–**C**) *Cuscuta pentagona*. (**D**–**F**) *Perilla frutescens* var. *frutescens*. (**G**–**I**) *P. frutescens* var. *crispa*. (**I**) Detailed glandular trichomes.

Observing the seeds using scanning electron microscopy revealed remarkable variation in testa surface patterns (Figure 3 and Figure 4, Table 2). Therefore, we categorized seeds into three major types based on qualitative characteristics such as ornamentation, epidermal cell pattern, cell wall shape, hilum, and hairiness. The epidermal cell outline was either isodiametric or elongated, while the boundary of the anticlinal cell wall was straight or sinuate and relief of the boundary was channeled or raised. The curvature of the outer periclinal cell walls was either concave or convex. The epidermal cell diameter ranged from 11.0–99.2 μm among all studied species, with *C. australis* having the smallest cell (average 20.8 μm) and *C. japonica* having the largest (average 70.8 μm; Table 2).

Besides *C. japonica,* all *Cuscuta* seeds displayed type I reticulate (net-like) ornamentation with isodiametric cell arrangement, a straight and raised anticlinal cell wall, and a concave outer periclinal cell wall (Figure 3A,B,D,E,G,H, and Figure 4A,B). *C. japonica* seeds displayed type II rugulate (puzzle-like) ornamentation with elongated cell arrangement, a straight and channeled anticlinal cell wall, and a convex outer periclinal cell wall (Figure 3J,K). *P. frutescens* vars. *frutescens* and *crispa* seeds showed type III rugose-colliculate ornamentation, a anticlinal cell wall, and a convex periclinal cell wall (Figure 4D,E,G,H). These seeds were channeled divided into subtypes based on anticlinal cell wall curvature: type III-1, *P. frutescens* var. *frutescens,* straight cell walls (Figure 4E); type III-2, *P. frutescens* var. *crispa,* sinuate cell walls (Figure 4H). Only the hilum surfaces of type III seeds were multicellular with stalked glandular trichomes (Figure 4F,I, Table 2).

### 2.4. Identification Key

Based on our general morphological and micromorphological analyses of CS and two adulterant seed types, we developed a key for the accurate identification of seeds according to shape, cross-sectional shape, the presence or absence of hairs on the hilum, embryo exudation, and combined micromorphological characteristics, such as testa ornamentation, epidermal cell pattern, and anticlinal and periclinal cell wall shape, as described below:

1. Seeds ovoid in shape; triangular cross-section; glabrous on hilum; rugulate and reticulate testa ornamentation ------------------------------------------------------------------------------------------------------------------------------------------------------------------ 2
1′. Seeds globose to subglobose in shape; circular to broadly triangular in cross-section; glandular hairs on hilum; rugose-colliculate testa ornamentation ----------------------------------------------------------------------------------------------------------------------------------------------------------------- 3
2. Embryo exudation in boiling water ------------------------------------------------------------------------------------------------------------------- 4
2′. No embryo exudation in boiling water ---------------------------------------------------- *Cuscuta pentagona*
3. Straight anticlinal cell wall boundary ------------------------------------- *Perilla frutescens* var. *frutescens*
3′. Sinuate anticlinal cell wall boundary ------------------------------------------- *Perilla frutescens* var. *crispa*
4. Seeds 2.17–3.26 × 1.57–2.78 mm; elliptic hilum; elongated epidermal cell outline; rugulate testa ornamentation; channeled anticlinal cell wall boundary; convex outer periclinal cell wall ----------------------------------------------------------------------------------------------------- *Cuscuta japonica*
4′. Seeds 1.17–1.82 × 1.04–1.46 mm; circular-ovate hilum; isodiametric epidermal cell outline; reticulate testa ornamentation; raised anticlinal cell wall boundary; concave outer periclinal cell wall ------------------------------------------------------------------------------ *Cuscuta australis* and *C. chinensis*

### 2.5. Comparision of rbcL Using Cuscuta

**Figure 5:**
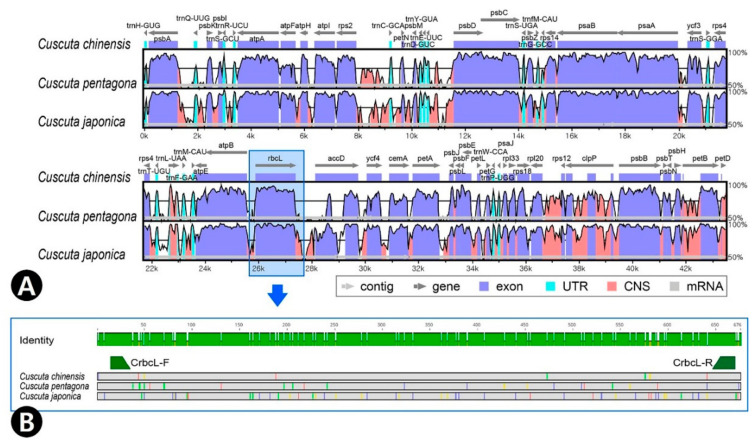
Schematic diagram of *Cuscuta* chloroplast genomes and novel DNA barcode primer design using *rbcL*. (**A**) Comparative analysis of the chloroplast genomes of three *Cuscuta* species using mVISTA, with the *C. japonica* chloroplast genome as the reference. Blue block, conserved genes; sky-blue blocks, tRNA and rRNA genes; red blocks, conserved non-coding sequences (CNSs); white blocks, polymorphic regions among the three *Cuscuta* species. A 50% identity cut-off was used for the plots. The *Y*-axis represents 50–100% identity. (**B**) Green arrows indicate novel DNA barcode primers. Colored regions indicate variable sequences in the three *Cuscuta* species.

To evaluate the *rbcL* gene as a potential DNA barcode to discriminate *Cuscuta* species and to test primer universality, we compared the whole chloroplast genome sequences of three *Cuscuta* species using the mVISTA program (Figure 5A). The *Cuscuta* chloroplast genomes were downloaded from Genbank (*C. chinensis*: MH780079; *C. pentagona*: NC_039759; *C. japonica*; MH780080). As the *rbcL* gene length varied in *C. chinensis* (1443 bp), *C. pentagona* (1446 bp), and *C. japonica* (1497 bp), we aligned their *rbcL* sequences and found that the region from 190 to 846 bp was relatively variable for species identification, with approximately 89–95% similarity. The flanking region is conserved and hence primers were designed from those regions. Therefore, we selected a 676 bp sequence from this region to design the forward and reverse primers (Figure 5B).

We confirmed the utility of the *rbcL* gene as a DNA barcode and developed novel DNA barcode primers using *rbcL*-generated marker sequences (Table 3). The *Cuscuta* subgenus *Monogynella* including *C. chinensis* and *C. pentagona* chloroplast genomes showed high sequence variability and lacked the *matK* gene, which is a universal DNA barcode region. Consequently, it is difficult to distinguish between *Cuscuta* species using universal DNA barcodes; however, the developed primer (CrbcL) had good discriminatory efficiency for *Cuscuta* species.

### 2.6. Comparision of ITS and rbcL Using Cuscuta and Perilla

To distinguish between the four *Cuscuta* species and two species of *P. frutescens*, we performed DNA barcode analysis using the nuclear rDNA ITS and *rbcL* regions. ITS sequences for *P. frutescens* var. *frutescens* and var. *crispa* were downloaded from Genbank (1: FJ513160; 2: KP644065; 3: KT220688; 4: KX397889; 5: MG223657; 6: MG224544; 7: KY624981). Similarly, the sequence data were obtained for 15 samples among the four *Cuscuta* species and aligned (Table 4). The ITS alignment region was 798 bp long in four *Cuscuta* species and 809 bp long in all studied taxa including *P. frutescens*. The *rbcL* alignment region was 656 bp long in all studied taxa. Among all taxa, there were 287 (35.5%) parsimony-informative sites in ITS and 102 (15.5%) in *rbcL*, whereas the ITS region was more variable than the *rbcL* region (ITS nucleotide diversity Pi = 0.17662; *rbcL* Pi = 0.06212). Intra-species variation in the four *Cuscuta* species was shown 0–3.4% in ITS, but not found in *rbcL*. Nine ITS haplotypes were detected in *C. pentagona* and two in *C. japonica*, indicating ambiguous species identification; however, only one of the four *Cuscuta* species shared a *rbcL* haplotype with *P. frutescens*. Thus, *rbcL* is more suitable for *Cuscuta* species identification than ITS, allowing complete discrimination of all four *Cuscuta* species.

### 2.7. Phylogenetic Analysis

**Figure 6:**
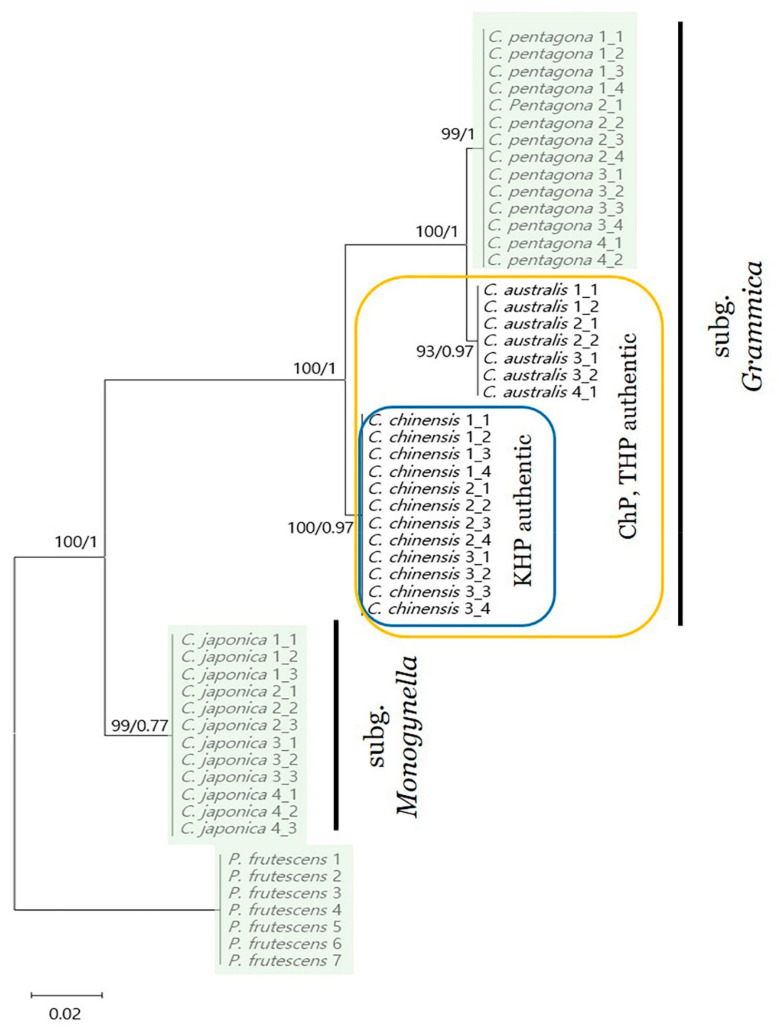
Phylogenetic analysis of five species using the maximum likelihood (ML) and Bayesian inference (BI) methods with *Cuscuta rbcL* sequences and *Perilla frutescens rbcL* sequences downloaded from Genbank as outgroups (1: FJ513160; 2: KP644065; 3: KT220688; 4: KX397889; 5: MG223657; 6: MG224544; 7: KY624981). ML topology is shown with bootstrap (BS) values (%) and BI posterior probability (PP) values at each node. ChP, Pharmacopoeia of the China; KHP, Korean Herbal Pharmacopoeia; THP, Taiwan Herbal Pharmacopeia. Green shaded species indicate adulterants of CS.

Finally, we analyzed the phylogenetic relationships between the *rbcL* sequences of four *Cuscuta* and one *P. frutescens* species using the maximum likelihood (ML) and Bayesian inference (BI) methods. All species clustered into monophyletic groups; however, *C. australis* and *C. pentagona* were more closely related to the other species (Figure 6 and Supplementary Materials Figure S1). This phylogeny could be further separated into two distinct clusters, one of which contained *C. australis*, *C. pentagona*, and *C. chinensis* with 100% bootstrap (BS) values, whereas the other only contained *C. japonica*. Phylogenetic analysis using ITS indicated an ambiguous node in both ML and BI trees (Figures S2 and S3): despite analyzing the same *C. pentagona* sequences, its phylogenetic position was varied and complex. However, *rbcL* clearly classified monophyletic relationships for each *Cuscuta* species.

## 3. Discussion

The increasing pharmacological and clinical importance of herbal medicine has resulted in issues related to misidentification and failed quality control. In this study, we used three different approaches, namely morphological and micromorphological analyses with statistical analysis and DNA barcoding, to accurately identify the widely consumed medicinal seed, CS. In addition, we created an effective identification key from the results of microscopic analysis to distinguish authentic medicinal seeds from adulterants and designed a specific and sensitive novel DNA barcode primer for the *rbcL* gene

### 3.1. Morphology-Based Identification

CS are small seeds that are difficult to distinguish from adulterants using the naked eye; in particular, three congeneric species (*C. australis*, *C. japonica*, and *C. pentagona*) in Korea have a very similar external seed morphology [58]. According to a dispensatory on the visual and organoleptic examination of herbal medicines, testa color and hilum position are important characteristics for authenticating CS [36]. In this study, we found that all *Cuscuta* seeds shared the same hilum position and that, although *C. australis* seeds were somewhat dark and blackish brown, their color varied widely (Gr-Br to Bl; Table 1), thus limiting the utility of these key characteristics. Ji et al. [49] described *C. australis* and *C. chinensis* seeds as having a “vomiting thread shape” and *C. japonica* seeds as “not vomiting thread shape” when put in water. However, we consistently observed embryo exudation (“vomiting thread”) in all *Cuscuta* seeds except for *C. pentagona* when placed into boiling water. The seed coat consists of three layers: the outer epidermis, two different palisade layers, and an inner multi-parenchymal layer. In most *Cuscuta* seeds, the endosperm surrounds the coiled embryo [59]; therefore, we hypothesize that embryo exudation is associated with seed coat thickness and the degree of endosperm embedding. Interestingly, the *C. campestris* embryo is only embedded in the endosperm in small regions [59], suggesting that *C. pentagona* may display a different internal structure to other *Cuscuta* species. Further comparative ultrastructural studies are required to evaluate the seed coat stratification and internal structure of seeds from different *Cuscuta* species.

### 3.2. Micromorphology-Based Identification

Our previous study suggested that the morphological characteristics of reproductive organs, particularly testa ornamentation, can be used to distinguish between *C. chinensis* and *C. japonica* [60]. Recently, the availability of detailed seed micromorphology has provided valuable diagnostic characteristics for authenticating small medicinal seeds [51,52] and taxonomic identification [61,62]. In this study, micromorphological analysis using a scanning electron microscope revealed that *Cuscuta* seeds including CS (types I, II, and glabrous hilum) are easily distinguishable from the seeds of adulterant *Perilla* species (type III and glandular hairs on hilum). In addition, the two types of testa ornamentation observed were consistent with subgenus classification [42]; *C. australis*, *C. chinensis*, and *C. pentagona* (subgenus *Grammica*, type I seeds) and *C. japonica* (subgenus *Monogynella*, type II seeds), suggesting that testa ornamentation may be stable at the subgenus level. Although seed micromorphology was a useful diagnostic characteristic, *C. australis* and *C. chinensis* could not be clearly distinguished and were difficult to differentiate using morphology or statistical analysis. Therefore, multilateral approaches including morphology with statistical analysis and DNA barcoding are required to accurately identify medicinal materials [53].

### 3.3. DNA Barcoding-Based Identification

Since 2010, the definition of CS in the Chinese Pharmacopoeia has changed to include both *C. chinensis* and *C. australis* [19]; however, quality control remains a major problem for CS due to the morphological similarity of these species, as *C. australis* and *C. chinensis* have significantly different chemical constituents [63]. To improve the pharmacological and clinical activities of CS, it is therefore important that these two similar medicinal seeds can be accurately identified. Molecular identification based on DNA barcoding is an efficient and accurate tool that has been used to authenticate medicinal species [55,56,57]; however, the method has limitations when using universal chloroplast DNA primers in closely related species [64,65]. As such, it is preferable to design species-specific primers or use next generation sequencing (NGS) and NGS-based DNA-metabarcoding to overcome this limitation of DNA-based analysis [66,67]. In this study, we accurately identified four *Cuscuta* species and adulterant *Perilla* species using novel genus-specific DNA barcode primers (CrbcL-F, CrbcL-R). Moreover, phylogenetic analysis yielded a phylogeny that was consistent with previous studies [42,48].

## 4. Materials and Methods

### 4.1. Plant Materials

Authentic species of CS (*C. chinensis*) and three congeneric species (*C. australis, C. japonica,* and *C. pentagona*) were collected from natural populations in Korea during the fruiting season (July 2016 to May 2019). All samples collected in this study were identified based on their macroscopic morphology and microscopic characteristics by two authors (Sungyu Yang and Jun-Ho Song) using relevant literature [38,39,42,58,68,69]. Commercial CS was purchased in medicinal markets from commercial suppliers (Kwang Myung Dang Co., Ulsan, Korea). The identity of the commercial CS (manufacturer’s No. K0412050015KE15) was carefully confirmed under a stereomicroscope (Olympus SZX16, Olympus, Tokyo, Japan). Adulterant seeds from *P. frutescens* var. *frutescens* and *P. frutescens* var. *crispa* were sampled from voucher specimens. The morphological and micromorphological characteristics of mature seeds from all species were analyzed. To ensure consistency, at least two samples were examined for each species. All seed samples were deposited in the Korean Herbarium of Standard Herbal Resources at the Korea Institute of Oriental Medicine (KIOM), Naju, Korea. Detailed information about the plant materials used in this study is summarized in Table 5, including the collection site, collection date, and voucher number of specimens deposited in the KB and KIOM.

### 4.2. General Morphology and Embryo Exudation

Twenty seeds per species (a total of 140 seeds) were measured and subjected to optical observation. Seed length, width, and the length/width ratio were measured using a digital vernier caliper (CD-15CP, Mitutoyo, Kawasaki, Japan). Shape, cross-sectional shape, color, hilum hair, and embryo exudation were observed using a stereomicroscope and images captured using a digital camera (Olympus DP21, Olympus, Tokyo, Japan). To observe embryo exudation, seeds were placed into boiling water for 10 min. Seed color was determined according to the Royal Horticultural Society Mini-color Chart [70].

### 4.3. Statistic Analysis

To verify whether four quantitative variables (seed length, seed width, L/W, and epidermal cell diameter) grouped the species, principal component analysis (PCA) was performed using PC-ORD version 5.31 [71].

### 4.4. Micromorphology

To fully dry the samples, the seeds were placed in silica gel desiccators for 10 days to remove any moisture. For micromorphological observation, seeds were directly mounted on aluminum stubs using a double sided adhesive conductive carbon disk (05073-BA, SPI Supplies, West Chester, PA, USA). The stubs were coated with gold using a sputter coater (208HR, Cressington Scientific Instruments Ltd., Watford, UK) and testa were observed using a low voltage field emission scanning electron microscope (JSM-7000F, JEOL, Tokyo, Japan) at an accelerating voltage of 5–10 kV and a working distance of 10–13 mm. The terminology for seed micromorphology followed that of Barthlott [72,73].

### 4.5. Sequence Analysis

DNA was extracted from all seed samples using a DNeasy Plant Mini kit (Qiagen, Valencia, CA, USA) according to the manufacturer’s instructions. Genomic DNA (20 ng) was amplified in a 20-µL PCR mixture (Solg^TM^ 2X Taq PCR smart mix 1, Solgent, Daegeon, Korea) with 10 pmol of each primer (Bioneer, Daejeon, Korea). The ITS region was amplified using ITS1 (TCC GTA GGT GAA CCT GCG G) and ITS4 (TCC TCC GCT TAT TGA TAT GC) primers, as described previously [74]. The conserved *rbcL* region in chloroplast genomes (*C. chinensis*: MH780079; *C. pentagona*: NC_039759; *C. japonica*; MH780080) was detected using mVISTA [75]. CrbcL primers were designed using Geneious Prime (Biomatters, Auckland, New Zealand) and tested by PCR amplification with 20 ng of genomic DNA from 15 *Cuscuta* samples in a 20 μL PCR mixture with 10 pmol of CrbcL primers using a Pro Flex PCR system (Applied Biosystems, Waltham, MA, USA) with the following parameters: initial denaturation at 95 °C for 2 min; 35 cycles at 95 °C for 50 s, 60 °C for 50 s, and 72 °C for 50 s; final extension at 72 °C for 5 min. The PCR products were separated on a 2% agarose gel for 40 min at 150 V. Each PCR product was isolated using a gel extraction kit (Qiagen), subcloned into a pGEM-T Easy vector (Promega, Madison, WI, USA), and sequenced using a DNA sequence analyzer (ABI 3730, Applied Biosystems Inc., Foster City, CA, USA). The newly sequenced chloroplast genome sequences in this study were deposited in the NCBI GenBank database under the accession numbers MT982734-MT982740, MT982754-MT982757, MT982776-MT982784, MT982792-982799, MT984385-MT984395, and MT998850-MT998863.

### 4.6. Comparative Analysis and Phylogenetic Analysis

The newly sequenced ITS and *rbcL* sequences of 15 samples from four *Cuscuta* species, eight *Perilla* ITS sequences, and seven *rbcL* sequences from Genbank were obtained and aligned using Multiple Alignment using Fast Fourier Transform. Gaps in the alignment were stripped using BioEdit [76]. DNA SP 6 [77] was used to calculate nucleotide variability (Pi), variable sites, and haplotypes. Phylogenetic analysis was carried out using a best-fit model based on Akaike Information Content using JModeltest V2.1.10 [78]. The GTR + I model was applied to ITS sequences (Table S1) and CrbcL (Table S2). ML analysis was performed using MEGA 6 [79] and branch support was calculated with 1000 bootstrap replicates. BI analysis was carried out using MrBayes 3.2.2 [80] with the following settings: two independent Markov Chain Monte Carlo runs performed for one million generations with samples every 1000 generations, the first 25% of trees were discarded as a burn-in.

## 5. Conclusions

To more accurately identify and authenticate herbal medicines, a multilateral strategy combining macroscopic and microscopic techniques with statistical analysis and DNA barcoding is essential. This is the first comprehensive and integrated study to accurately identify medicinal CS and adulterant seeds. Morphology- and micromorphology-based analyses revealed that general shape, embryo exudation, hairiness, and testa ornamentation are useful characteristics for identifying and authenticating the studied species. Furthermore, we were able to completely discriminate between four *Cuscuta* species and two *Perilla* adulterants species using novel genus-specific DNA barcode primers. Therefore, our morphological and molecular data should allow the accurate identification and quality control of CS.

## Figures and Tables

**Table 1:** Seed morphological characteristics of original species of Cuscutae Semen, congeneric, and adulterant species.

Taxa	Shape ^a^	Cross-Sectional Shape ^b^	Length (mm)	Width (mm)	L/W	Color ^c^	Embryo ^d^
*Cuscuta australis* (1)	ovo	tri	1.17-(1.48)-1.72	1.06-(1.12)-1.24	1.08-(1.32)-1.56	Gr-Br (199A-B)	+
*Cuscuta australis* (2)	ovo	tri	1.25-(1.39)-1.53	0.98-(1.15)-1.34	1.06-(1.22)-1.43	Gr-Br (N199A-B), Bl (202A)	+
*Cuscuta* *chinensis*	ovo	tri	1.29-(1.56)-1.82	1.04-(1.25)-1.46	1.03-(1.24)-1.50	Br (200A-D), Gr-Br (199A-B)	+
*Cuscuta* *japonica*	ovo	tri	2.17-(2.71)-3.26	1.57-(2.16)-2.78	1.05-(1.27)-1.57	Gr-Br (N199C-D), Bl (203)	+
*Cuscuta* *pentagona*	ovo	tri	1.36-(1.65)-1.87	1.09-(1.32)-1.47	1.03-(1.24)-1.48	Br (200A-D)	−
*Perilla frutescens* var. *frutescens*	glo to subglo	cir to broadly tri	2.19-(2.40)-2.83	1.79-(2.00)-2.28	1.06-(1.20)-1.39	Gr-Br (199A-B, N199A-C)	−
*Perilla frutescens* var. *crispa*	glo to subglo	cir to broadly tri	1.58-(1.97)-2.45	1.54-(1.87)-2.11	0.96-(1.05)-1.20	Gr-Br (199A-B, N199A-C)	−

^a^ glo, globose; subglo, subglobose; ovo, ovoid. ^b^ cir, circular; tri, tri-angular. ^c^ Bl, black; Br, brown; Gr, gray. ^d^ Embryo exudation when the seeds were boiled in water for 10 min, −, no exudation; +, exudation.

**Table 2:** Seed micromorphological characteristics of original species of Cuscutae Semen, congeneric, and adulterant species.

Taxa	Ornamentation ^a^ (Type)	Cell Outline ^b^	Epidermal Cell Diameter (μm)	Anticlinal Cell Wall ^c^	Periclinal Cell Wall ^d^	Hilum Shape ^e^	Hairy ^f^
*Cuscuta australis* (1)	ret (I)	iso	11.0-(21.5)-27.4	str, rsd	ccv	cir-ova	-
*Cuscuta australis* (2)	ret (I)	iso	15.7-(20.2)-26.3	str, rsd	ccv	cir-ova	-
*Cuscuta* *chinensis*	ret (I)	iso	19.5-(25.4)-35.0	str, rsd	ccv	cir-ova	-
*Cuscuta* *japonica*	rug (II)	elg	49.3-(70.8)-99.2	str, chn	cvx	ell	-
*Cuscuta* *pentagona*	ret (I)	iso	15.2-(19.5)-24.3	str, rsd	ccv	cir-ova	-
*Perilla frutescens* var. *frutescens*	rgs-col (III-1)	elg	32.6-(40.5)-53.0	str, chn	cvx	cir	gl
*Perilla frutescens* var. *crispa*	rgs-col (III-2)	elg	20.8-(24.8)-39.8	sin, chn	cvx	cir	gl

^a^ col, colliculate; ret, reticulate; rgs, rugose; rug, rugulate. ^b^ elg, elongated; iso, isodiametric. ^c^ chn, channeled; sin, sinuate; str, straight; rsd, raised. ^d^ ccv, concave; cvx, convex. ^e^ cir, circular; ell, elliptic; ova, ovate. ^f^, absent; gl, present gland on hilum.

**Table 3:** Novel DNA barcode primers for *Cuscuta* species.

Primer Name	Primer Sequence (5′ –> 3′)	Position
CrbcL-F	GGTACATGGACAACTGTGTGG	*rbcL*
CrbcL-R	TGAGCCAAAGAAGTATTTGCAGTG	

**Table 4:** Comparative analysis of DNA barcodes for four *Cuscuta* species and two *Perilla frutescens*.

Region	Species	Alignment Length	Parsimony Informative Site		Variable site		Nucleotides Diversity (Pi)	No. of Haplotypes
			Number	%	Number	%		
ITS	Four *Cuscuta*	798	202	25.3%	205	25.7%	0.08883	13
	*C. pentagona*	784	25	3.2%	27	3.4%	0.01661	9
	*C. australis*	784	0	0.0%	0	0.0%	0	1
	*C. chinensis*	784	0	0.0%	0	0.0%	0	1
	*C. japonica*	793	0	0.0%	1	0.1%	0.00042	2
	*P. frutescens*	615	5	0.8%	0	0.0%	0.00461	4
	Total	809	287	35.5%	289	35.7%	0.17662	17
*rbcL*	Four *Cuscuta*	656	70	10.7%	70	10.7%	0.04645	4
	*C. pentagona*	656	0	0.00%	0	0.0%	0	1
	*C. australis*	656	0	0.00%	0	0.0%	0	1
	*C. chinensis*	656	0	0.00%	0	0.0%	0	1
	*C. japonica*	656	0	0.00%	0	0.0%	0	1
	*P. frutescens*	656	0	0.00%	0	0.0%	0	1
	Total	656	102	15.5%	102	15.50%	0.06212	5

**Table 5:** List of plant species used in this study along with the collection site, collection date, and voucher number.

Scientific Name	Collection Site (Commercial Suppliers)	Collection Date	Voucher No.
Cuscuta australis (1) ^†,‡^	Hapcheon-gun, Gyeongsangnam-do, Korea	5 August 2017	NIBRVP0000652021 ^M^
	Hapcheon-gun, Gyeongsangnam-do, Korea	20 August 2019	KIOM201901022449 ^M^
	Hapcheon-gun, Gyeongsangnam-do, Korea	20 August 2019	KIOM201901022450 ^B,M^
*Cuscuta australis* (2) ^†,‡^	China ^∥^ (Kwang Myung Dang Co., Ulsan, Korea)	12 May 2017	2-19-0369 ^M^
*Cuscutachinensis* *^,^^†^	Hallim-eup, Jeju-si, Jeju, Korea	29 August 2016	KIOM201601017927 ^B,M^
	Hallim-eup, Jeju-si, Jeju, Korea	29 August 2016	KIOM201601017928 ^B,M^
	Yeongcheon-si, Gyeongsangbuk-do, Korea	2 August 2016	NIBRGR0000431983 ^B,M^
*Cuscuta* *japonica* ^‡^	Danyang-gun, Chungcheongbuk-do, Korea	28 July 2016	KIOM201701018784 ^B,M^
	Bonghwa-gun, Gyeongsangbuk-do, Korea	25 August 2016	MBC_KIOM-2016-279 ^B,M^
	Gujwa-eup, Jeju-si, Jeju, Korea	29 August 2016	NIBRVP0000646517 ^M^
	Jeongseon-gun, Gangwon-do, Korea	13 October 2016	NIBRVP0000591879 ^M^
*Cuscuta* *pentagona*	Aewol-eup, Jeju-si, Jeju, Korea	29 August 2016	KIOM201601017931 ^B,M^
	Yuseong-gu, Daejeon, Korea	27 July 2016	KIOM201701018523 ^B,M^
	Jinan-gun, Jeollabuk-do, Korea	3 October 2016	MBC_KIOM-2016-365 ^B^
	Danyang-gun, Chungcheongbuk-do, Korea	28 July 2016	KIOM201701018786 ^M^
*Perilla frutescens* var. *frutescens* ^§^	Inje-gun, Gangwon-do, Korea	8 October 2010	NIBRVP0000272211 ^M^
*Perilla frutescens* var. *crispa* ^§^	Buan-gun, Jellabuk-do, Korea	13 October 2006	VP-CNNU-356073-5524 ^M^
	Daejeong-eup, Seogwipo-si, Jeju, Korea	29 August 2016	KOSPVP0000238074 ^M^

* Official species designated as materials of CS in the Korean Herbal Pharmacopoeia. ^†^ Official species designated as materials of CS in both Pharmacopoeia of the People’s Republic of China and Taiwan Herbal Pharmacopeia. ^‡^ Official species designated as materials of CS in the Pharmacopoeia of Democratic People’s Republic of Korea. ^§^ Adultrants. ^∥^ Commercial CS were purchased from the medicinal material market. ^B^ DNA barcoding analysis; ^M^ morphological analysis.

## Supplementary Materials

The following are available online at https://www.mdpi.com/2223-7747/9/11/1410/s1. Figure S1: Phylogenetic analysis of five species. The phylogenetic tree was constructed from *Cuscuta* species with the Bayesian inference (BI) method. The *rbcL* sequences of *Perilla frutescens* were downloaded from GenBank and used as outgroups. BI posterior probability (PP) values at each node. Figure S2: Phylogenetic analysis of five species. The phylogenetic tree was constructed from *Cuscuta* species with the maximum likelihood (ML) method. The ITS sequences of *Perilla frutescens* were downloaded from GenBank and used as outgroups. ML posterior probability (PP) values at each node. Figure S3: Phylogenetic analysis of five species. The phylogenetic tree was constructed from *Cuscuta* species with the Bayesian inference (BI). The ITS sequences of *Perilla frutescens* were downloaded from GenBank and used as outgroups. BI posterior probability (PP) values at each node. Table S1: Selection of the best-fitting substitution model for ITS using jModelTest. Table S2: Selection of the best-fitting substitution model for *rbcL* using jModelTest.
