# Supplementary material for: An Integrated Approach for Efficient and Accurate Medicinal Cuscutae Semen Identification
Source: Plants (Basel). 2020 Oct 22;9(11):1410. doi: 10.3390/plants9111410 (PMC7690581; doi:10.3390/plants9111410)
Supplement: Supplementary file 1 [file plants-09-01410-s001.pdf]

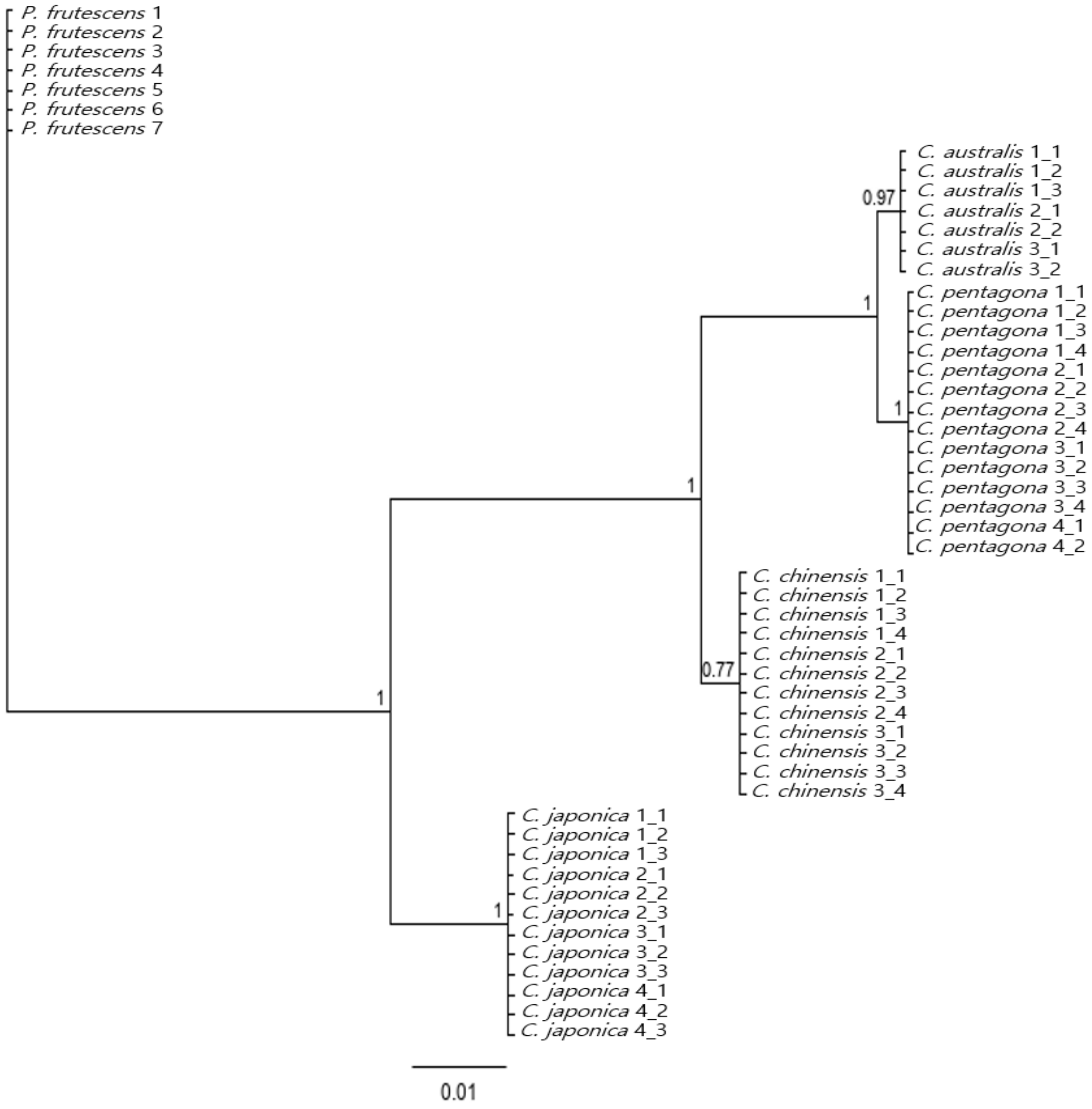


**Figure S1.** Phylogenetic analysis of five species. The phylogenetic tree was constructed from *Cuscuta* species with the Bayesian inference (BI) method. The *rbcL* sequences of *Perilla frutescens* were downloaded from GenBank and used as outgroups. BI posterior probability (PP) values at each node.

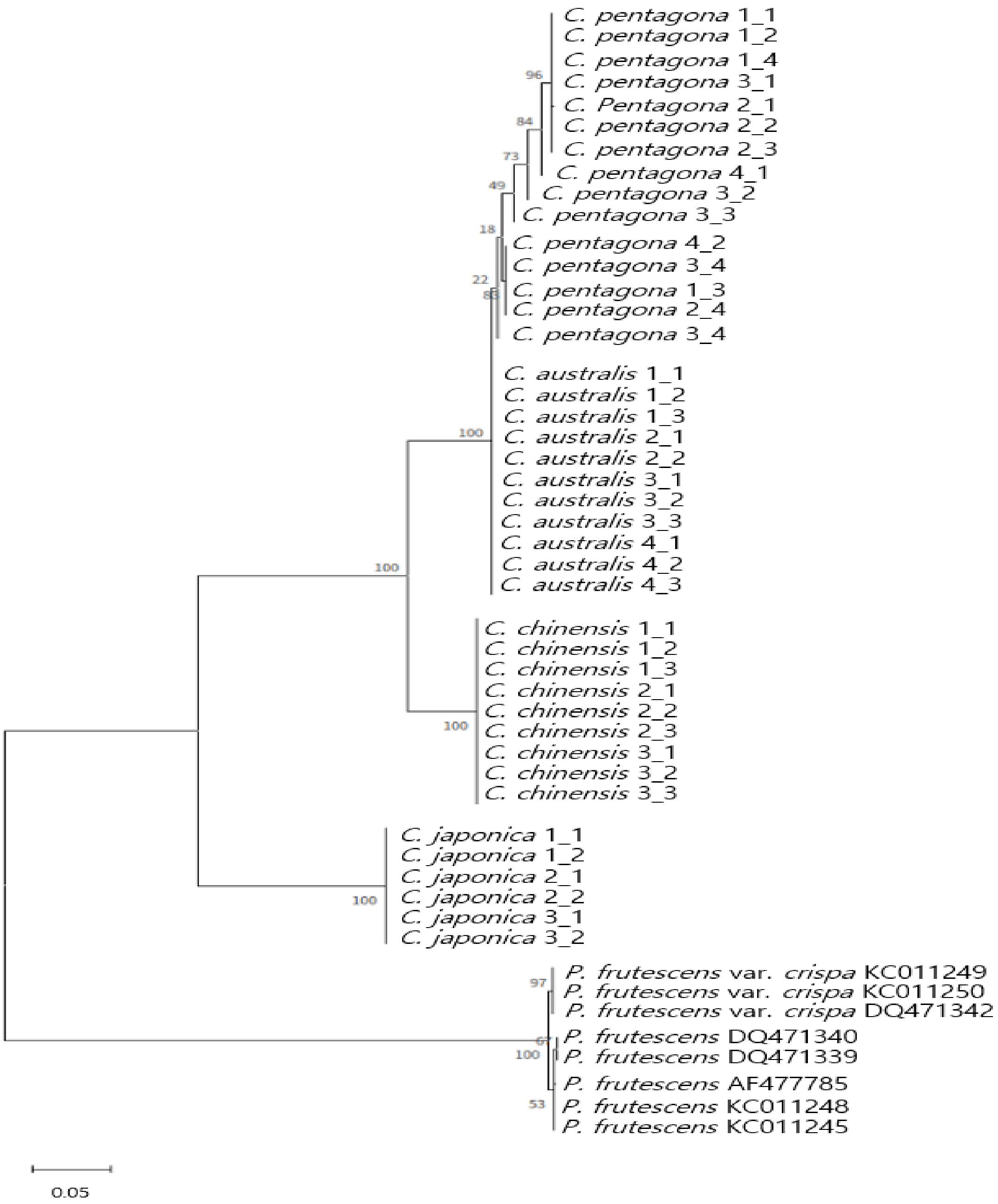


**Figure S2.** Phylogenetic analysis of five species. The phylogenetic tree was constructed from *Cuscuta* species with the maximum likelihood (ML) method. The ITS sequences of *Perilla frutescens* were downloaded from GenBank and used as outgroups. ML posterior probability (PP) values at each node.

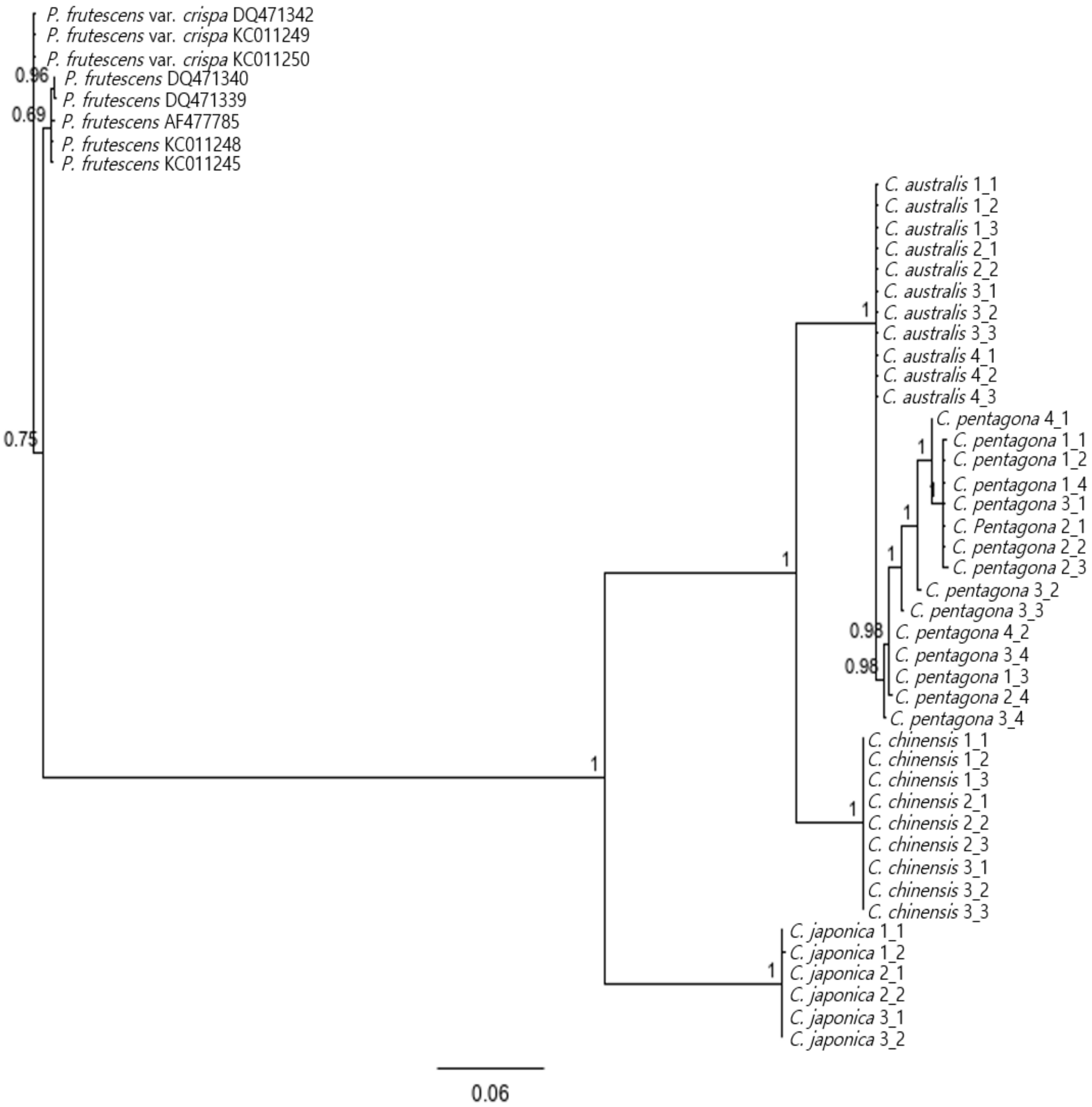


**Figure S3.** Phylogenetic analysis of five species. The phylogenetic tree was constructed from *Cuscuta* species with the Bayesian inference (BI). The ITS sequences of *Perilla frutescens* were downloaded from GenBank and used as outgroups. BI posterior probability (PP) values at each node.

**Table S1.** Selection of the best-fitting substitution model for ITS using jModelTest.

| | Model | f(a) | f(c) | f(g) | f(t) | kappa | titv | Ra | Rb | Rc | Rd | Re | Rf | pInv | Gamma |
|---|---|---|---|---|---|---|---|---|---|---|---|---|---|---|---|
| AIC | GTR+I | 0.22 | 0.28 | 0.27 | 0.21 | 2.47 | 1.23 | 0.77 | 2.73 | 1.41 | 0.60 | 1.63 | 1 | 0.25 | 1.36 |

| Model | −lnL* | K | AIC | delta | weight | cumWeight |
|---|---|---|---|---|---|---|
| GTR+I | 3525.119 | 123 | 7296.239 | 0 | 0.188444 | 0.188444 |
| TIM3+I | 3527.159 | 121 | 7296.317 | 0.0782 | 0.181218 | 0.369661 |
| TIM3+G | 3527.24 | 121 | 7296.48 | 0.24118 | 0.167036 | 0.536697 |
| GTR+G | 3525.316 | 123 | 7296.633 | 0.39384 | 0.15476 | 0.691457 |
| TIM3+I+G | 3526.67 | 122 | 7297.339 | 1.10066 | 0.108687 | 0.800144 |
| GTR+I+G | 3524.679 | 124 | 7297.357 | 1.1182 | 0.107738 | 0.907882 |
| TIM2+G | 3529.464 | 121 | 7300.927 | 4.68836 | 0.018077 | 0.925958 |
| TIM2+I | 3529.584 | 121 | 7301.169 | 4.93014 | 0.016018 | 0.941976 |
| TIM2+I+G | 3529.029 | 122 | 7302.059 | 5.81998 | 0.010266 | 0.952242 |
| TrN+G | 3531.407 | 120 | 7302.813 | 6.57424 | 0.00704 | 0.959283 |
| TrN+I | 3531.679 | 120 | 7303.358 | 7.11896 | 0.005362 | 0.964645 |
| TVM+I | 3529.783 | 122 | 7303.566 | 7.32746 | 0.004831 | 0.969476 |
| TPM3uf+I | 3531.825 | 120 | 7303.649 | 7.41048 | 0.004635 | 0.97411 |
| TPM3uf+G | 3531.945 | 120 | 7303.89 | 7.65072 | 0.00411 | 0.97822 |
| TrN+I+G | 3531.047 | 121 | 7304.094 | 7.85524 | 0.003711 | 0.981931 |
| TVM+G | 3530.082 | 122 | 7304.163 | 7.92464 | 0.003584 | 0.985515 |
| TIM1+G | 3531.371 | 121 | 7304.742 | 8.50342 | 0.002683 | 0.988198 |
| TPM3uf+I+G | 3531.383 | 121 | 7304.767 | 8.52774 | 0.002651 | 0.990849 |
| TVM+I+G | 3529.42 | 123 | 7304.839 | 8.60036 | 0.002556 | 0.993406 |
| TIM1+I | 3531.654 | 121 | 7305.307 | 9.06824 | 0.002023 | 0.995429 |
| TIM1+I+G | 3531.013 | 122 | 7306.025 | 9.78662 | 0.001413 | 0.996842 |

*−lnL, negative log likelihood; K, number of estimated parameters; AIC, Akaike Information Criterion; delta, AIC difference; weight, AIC weight; cumWeight, cumulative AIC weight.

**Table S2.** Selection of the best-fitting substitution model for *rbcL* using jModelTest.

| | Model | f(a) | f(c) | f(g) | f(t) | kappa | titv | Ra | Rb | Rc | Rd | Re | Rf | pInv | Gamma |
|---|---|---|---|---|---|---|---|---|---|---|---|---|---|---|---|
| AIC | GTR+I | 0.29 | 0.18 | 0.22 | 0.29 | 2.58 | 1.25 | 1.76 | 2.16 | 0.62 | 1.49 | 3.76 | 1 | 0.851 | 0.851 |

| Model | −lnL* | K | AIC | delta | weight | cumWeight |
|---|---|---|---|---|---|---|
| GTR+I | 1495.75 | 111 | 3213.5 | 2.22032 | 0.052392 | 0.528477 |
| TrN+G | 1498.765 | 108 | 3213.529 | 2.24942 | 0.051635 | 0.580112 |
| TIM3+I+G | 1496.779 | 110 | 3213.558 | 2.27836 | 0.050893 | 0.631005 |
| GTR+G | 1495.915 | 111 | 3213.829 | 2.5492 | 0.044448 | 0.675452 |
| TPM3uf+I | 1498.975 | 108 | 3213.95 | 2.66998 | 0.041843 | 0.717295 |
| TPM3uf+G | 1499.138 | 108 | 3214.276 | 2.99584 | 0.035552 | 0.752847 |
| TIM1+I+G | 1497.603 | 110 | 3215.205 | 3.92536 | 0.022337 | 0.775184 |
| TIM2+I | 1498.634 | 109 | 3215.269 | 3.98858 | 0.021642 | 0.796825 |
| TIM2+G | 1498.651 | 109 | 3215.302 | 4.0215 | 0.021288 | 0.818114 |
| TPM1uf+I | 1499.729 | 108 | 3215.458 | 4.17826 | 0.019683 | 0.837797 |
| TrN+I+G | 1498.774 | 109 | 3215.548 | 4.26842 | 0.018816 | 0.856613 |
| TPM1uf+G | 1499.84 | 108 | 3215.679 | 4.39946 | 0.017623 | 0.874235 |
| GTR+I+G | 1495.915 | 112 | 3215.829 | 4.54926 | 0.016351 | 0.890586 |
| HKY+I | 1500.946 | 107 | 3215.891 | 4.611 | 0.015854 | 0.90644 |
| HKY+G | 1501.017 | 107 | 3216.034 | 4.7544 | 0.014757 | 0.921197 |
| TVM+I | 1498.049 | 110 | 3216.099 | 4.8188 | 0.014289 | 0.935486 |
| TPM3uf+I+G | 1499.138 | 109 | 3216.276 | 4.9959 | 0.013078 | 0.948565 |
| TVM+G | 1498.234 | 110 | 3216.468 | 5.18752 | 0.011883 | 0.960448 |
| TIM2+I+G | 1498.658 | 110 | 3217.316 | 6.03618 | 0.007774 | 0.968222 |

*−lnL, negative log likelihood; K, number of estimated parameters; AIC, Akaike Information Criterion; delta, AIC difference; weight, AIC weight; cumWeight, cumulative AIC weight.
